# A Toolbox of Potential Immune-Related Therapies for Inflammatory Cardiomyopathy

**DOI:** 10.1007/s12265-020-10025-4

**Published:** 2020-05-21

**Authors:** Ahmed Elsanhoury, Carsten Tschöpe, Sophie Van Linthout

**Affiliations:** 1 Berlin Institute of Health Center for Regenerative Therapies (BCRT), Charité – Universitätsmedizin Berlin, Campus Virchow Klinikum (CVK), Föhrerstrasse 15, 13353 Berlin, Germany; 2 German Center for Cardiovascular Research (DZHK), Partner site Berlin, Berlin, Germany; 3 Department of Cardiology, Charité – Universitätsmedizin Berlin, Campus Virchow Klinikum (CVK), Berlin, Germany

**Keywords:** Inflammatory cardiomyopathy, Immunosuppression, Immunomodulation

## Abstract

Myocarditis is a multifactorial disorder, characterized by an inflammatory reaction in the myocardium, predominantly triggered by infectious agents, but also by antigen mimicry or autoimmunity in susceptible individuals. Unless spontaneously resolved, a chronic inflammatory course concludes with cardiac muscle dysfunction portrayed by ventricular dilatation, clinically termed inflammatory cardiomyopathy (Infl-CM). Treatment strategies aim to resolve chronic inflammation and preserve cardiac function. Beside standard heart failure treatments, which only play a supportive role in this condition, systemic immunosuppressants are used to diminish inflammatory cell function at the cost of noxious side effects. To date, the treatment protocols are expert-based without large clinical evidence. This review describes concept and contemporary strategies to alleviate myocardial inflammation and sheds light on potential inflammatory targets in an evidence-based order.

## Introduction

Inflammation, a complex biological response to injury, infection, and exposed autoantigens, sometimes stirs in the myocardium. Myocardial inflammation is characterized by nonspecific symptoms like chest pain, arrhythmias, and heart failure signs of non-ischemic origin [1, 2]. The inflammatory reaction is most frequently associated with viral infections including coxsackievirus B3 (CVB3), adenoviruses, and active parvovirus B19 (B19V), whereas trypanosomes, bacteria, toxic substances, and autoantigens are other frequent etiologies [2, 3]. Myocarditis may spontaneously resolve without clinical footprints or remain active, instigating a chronic inflammatory course that culminates into inflammatory cardiomyopathy (Infl-CM), characterized by left ventricular (LV) dysfunction and heart failure or arrhythmias [1–4]. Cardiotropic microbes and cardiotoxins induce acute myocarditis via direct activation of the host immune system or via induction of myocyte necrosis and exposure of normally hidden antigens [2, 4]. Although in the acute phase, cardiac inflammation can be detected by cardiac magnetic resonance imaging, endomyocardial biopsy (EMB) analysis is the only diagnostic tool capable of identifying the underlying etiology of cardiac inflammation, allowing quantification of immune cell subtypes and microbial nucleic acid [2, 5–7].

The associated inflammatory processes are diverse and complex, including microbial or nonmicrobial inducers (e.g., alarmins, extracellular matrix fragments, and self-proteins), which activate immune sensors (e.g., inflammasomes, Toll-like receptors) and several kinds of mediators including cytokines, chemokines (e.g., CC-chemokine ligand (CCL)2 and CCL7), eicosanoids (e.g., prostaglandins), biogenic amines (e.g., histamine), and bioactive peptides (e.g., bradykinin) [8]. Being the major peripheral lymphatic reservoir of monocytes and filter of viruses, the spleen plays a major role in the development of Infl-CM. Monocytes from the spleen home to the heart (cardiosplenic axis), where they next contribute to tissue injury and cardiac remodeling [9–13].

Immunosuppressant agents like lympholytic, anti-proliferative agents and proliferation signal inhibitors are seen as potential therapies for myocardial inflammation, frequently indicated as off-label treatments or used in the context of clinical studies. For the moment, most clinical studies in this arena are investigator-initiated, and there is a lack of sufficient information concerning the clinical value of individual immunosuppressive agents. Etiology-based treatment protocols are needed for which EMB analysis subclassifies the patients to different strata [8]. There is no available consensus in general, due to the lack of large patient registries and randomized controlled trials, yet this review aims to sketch the contemporary strategies promised to counteract myocardial inflammation and to minimize its impact on the myocardium. The order of available strategies is built on the degree of clinical and experimental evidence, starting with (I) global immunosuppressive strategies antagonizing cellular and humoral immunity, followed by strategies, which (II) systemically modulate the immune response, (III) antagonize key inflammatory components, (IV) reduce myocardial wall stress via mechanical unloading, and (V) decrease mimic peptides-driven anti-cardiac autoimmunity via antibiotic therapy (Table 1).

**Table 1:** Overview of available immune-related strategies suggested for the treatment of inflammatory cardiomyopathy

Strategies	Working mechanism
Strategy I: Global immune system suppression	
Prednisolone plus azathioprine or cyclosporine A	Prednisolone: leukocyte and eicosanoids suppression. Azathioprine: depletion of activated lymphocytes and induction of antigen-specific tolerance. Cyclosporine: calcineurin inhibition
Mycophenolate mofetil	Selective T and B lymphocyte depletion
Rituximab	Selective B lymphocyte depletion
Methotrexate	Suppression of lymphocyte function
Sirolimus	Inhibition of mTOR signaling
Strategy II: Systemic modulation of the immune response	
Intravenous immunoglobulins	Buffering different pro-inflammatory responses. Aid in pathogen recognition and clearance
Interferon-ß	Regulation of cell-mediated immunity
Autoantibody therapies	Depletion of autoantibodies
Cannabidiol	Attenuation of different immune-mediated cardiotoxic processes via unknown mechanism(s)
Cell-based therapies	Induction of/reestablishing immune tolerance
Strategy III: Antagonizing key inflammatory components	
Colchicine	Suppression of neutrophils and NLRP3 inflammasome signaling
Anakinra or canakinumab	Antagonizing interleukin-1
Q-compounds	Antagonizing S100A8/S100A9 alarmins
Strategy IV: Reducing myocardial wall stress via mechanical unloading	
Strategy V: Decreasing mimic peptides-driven anti-cardiac autoimmunity via antibiotic therapy	

mTOR, mammalian target of rapamycin; NLRP3, nucleotide oligomerization domain (NOD)-, leucine-rich repeat (LRR)- and pyrin domain-containing protein 3

## Strategy I: Global Immune System Suppression

**Fig. 1:**
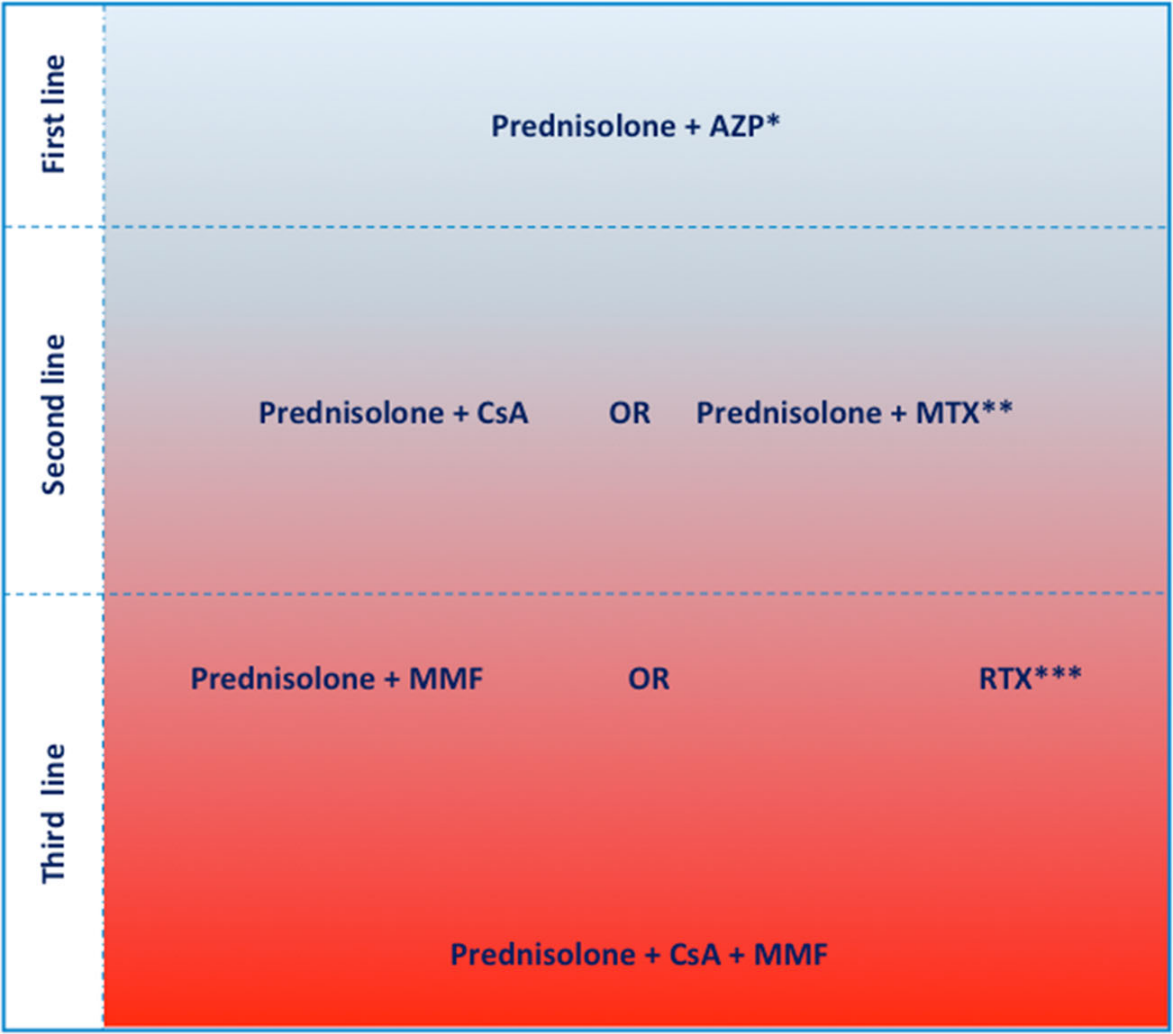
Empirical immunosuppression treatment options for endomyocardial biopsy-proven microbial-negative myocardial inflammation, based on reported clinical evidence and expert-opinions. Red gradient indicates higher risk of immunosuppression-associated side effects and poorer clinical experience. Different immunosuppressive combinations are clinically relevant in cases of drug-specific contraindications and/or severe side effects. AZP, azathioprine; MTX, methotrexate; RTX, rituximab; MMF, mycophenolate mofetil. *AZP is not recommended in cases with chronic liver disease. **Single-center experience, used in patients developing nephrotoxicity in response to CsA. ***Used in cases with EMB-proven persistent CD20+ B lymphocyte infiltrates

Immunosuppressive therapies are used to counteract a broad range of deleterious conditions attributed to exaggerated or inappropriate immune responses responsible for acute or chronic inflammation. Agents of this pharmacological class are known prescription medications for autoimmune disorders like rheumatoid arthritis and for transplant rejection prevention [14]. Besides, immunosuppressive therapies are indicated to counteract unrestrained inflammation that persists in the absence of an inflammatory trigger. Leukocytes mediate inflammation and tissue damage through the release of lysosomal enzymes, toxic hydroxyl radicals, and chemotactic cytokines that further activate the inflammatory cascade and recruit collagen-producing cells [15]. The use of immunosuppression-based strategies in the context of myocarditis and Infl-CM is only recommended if active infection is ruled out via EMB-based molecular diagnostics [2]. Empirical immunosuppression treatment options for EMB-proven microbial-negative myocardial inflammation, based on reported clinical evidence and expert-opinions, are discussed below and outlined in Fig. 1.

### Prednisolone, Azathioprine, and Cyclosporine

Prednisolone is a synthetic corticosteroid, able to dramatically reduce inflammation irrespective of its origin, owning to its diverse suppressive effects on leukocytes and inflammatory mediators. Prednisolone inhibits leukocyte extravasation and reduces macrophage-phagocytic functions and production of TNF-α, IFN-γ, IL-1, and IL-12. Besides, prednisolone has profound effects on eicosanoids via (1) inhibition of phospholipase A2, an enzyme responsible for the formation of arachidonic acid, the precursor of prostaglandins and leukotrienes, and (2) reduction in expression of cyclooxygenase enzyme (COX) isoform II, responsible for the synthesis of prostaglandins from arachidonic acid [16].

Azathioprine (AZP) is a prodrug metabolically activated to 6-mercaptopurine, which forms masquerade purine nucleotides, cytotoxic to activated lymphocytes. In addition, the prodrug is believed to induce antigen-specific tolerance by interfering with CD28 co-stimulatory signaling [16, 17].

Cyclosporine A (CsA) is a more potent, widely used immunosuppressant in organ transplantation and severe inflammatory disorders. This potent immunosuppressant forms a complex with a cytosolic immunophyllin, called cyclophilin, which upon formation inhibits calcineurin. Inhibition of calcineurin leads to a reduction in IL-2, IL-3, and IFN-γ transcription and T cell activity [16, 18]. The bioavailability of CsA varies among patients, which require individualized dose adjustment [16, 19]. CsA is known to increase the risk of lymphomas and other malignancies. Furthermore, it induces irreversible renal impairment, mandating close monitoring of renal function.

In lymphocytic myocarditis, prednisolone alone or in combination with AZP and/or CsA is the most frequently used immunosuppressive regimens. The outcomes of prednisolone/AZP combination therapy in virus-negative Infl-CM are largely favorable. A large retrospective controlled study including 90 patients per group (propensity score matched) demonstrated significant improvements in LV ejection fraction (EF) and heart transplantation-free survival in the group treated with prednisolone/AZP combination compared with the control group treated with standard heart failure medications [20]. The TIMIC study, which employed a prospective randomized, double-blind, placebo-controlled design demonstrated the efficacy of the combination therapy in virus-negative Infl-CM patients [21]. Favorable long-term outcomes were demonstrated in similar patients selected according to the ESC EMB-based diagnostic criteria [22]. Recently, Tschöpe et al. showed a therapeutic value for prednisolone/AZP combination in B19V DNA-positive myocarditis patients. The regimen downsized inflammation, yet, left B19V DNA copy number unaffected [23]. CsA combined with prednisolone is sometimes used to counteract severe cardiac muscle diseases including giant cell myocarditis, eosinophilic myocarditis, and cardiac sarcoidosis [7, 24]. CsA/prednisolone combination therapy was associated with EMB-proven improvement of myocarditis in infants with Infl-CM [25]. Cyclophilin, the pharmacological target of cyclosporine A, was found to be enhanced in EMB samples from Infl-CM patients [26] and to contribute to inflammation in a murine CVB3-induced myocarditis model, suggesting CsA as potential therapy [27]. Theoretically, tacrolimus, another calcineurin inhibitor, could be of therapeutic value. However, its use in myocarditis and Infl-CM patients is restrained, since the drug is alleged to contribute to cardiac toxicity, driving different forms of cardiomyopathies [28–31].

### Mycophenolate Mofetil

Mycophenolate mofetil (MMF), a prodrug of mycophenolic acid, which has immunosuppressive and anti-proliferative properties, is the drug of choice in solid organ transplant patients with refractory rejection [16, 32]. The active acid inhibits de novo synthesis of purines, leading to a nearly selective inhibition of DNA replication in T and B lymphocytes [16, 32–34]. Besides, MMF impedes leukocyte adhesion [16, 35]. Owing to multiple mechanisms of action, MMF abrogates both cellular and humoral immunity. In an experimental autoimmune myocarditis murine model, MMF has been shown to interfere with the disease development [36]. Another murine study observed an improvement in CVB3-induced myocarditis in MMF-treated mice [37]. De Luca et al. assessed the efficacy of MMF in a small cohort of virus-negative myocarditis patients who were intolerant/refractory to AZP. A 6-month treatment course improved cardiac function and reduced the mean LV end diastolic diameter together with the circulating markers troponin T and NT-proBNP [38]. A recent case report has presented the efficacy of triple immunosuppressive therapy comprising prednisolone/CsA and MMF in a cardiogenic shock case with recurrent giant cell myocarditis. The addition of MMF to the dual immunosuppressive therapy abrogated myocardial inflammation and allowed steroid dose reduction and full recovery [39]. In another case report, Tschöpe et al. revealed an increased presence of CD20+ B lymphocytes in lymphocytic myocarditis patients refractory to combined immunosuppressive therapy [40]. This phenotype is a good example for the requirement of personalized treatment and could be targeted with MMF. Whether MMF treatment is efficient to treat those patients has not been investigated so far.

### Rituximab

Rituximab (RTX) is a chimeric monoclonal antibody that binds to the CD20 protein on the B lymphocyte surface, mediating specific depletion of CD20+ B lymphocytes via apoptosis stimulation and complement-dependent/cellular-mediated cytotoxicity. Clinically, RTX is indicated for B non-Hodgkin’s lymphomas, chronic lymphocytic leukemia, and Wegener’s disease [16, 41, 42]. CD20+ B lymphocytes contribute to myocardial damage directly as well as via amplifying the effector functions of T lymphocytes and monocytes [43–47]. Notably, CD20+ B lymphocytes are resilient against steroid-based therapies [40, 48]. A case series described a subset of Infl-CM patients, who were refractory to combined steroid-based immunosuppression. The specific patients showed CD20+ B lymphocytic infiltrates in the EMB samples, which resolved upon RTX treatment, mirrored with clinical improvement [40].

### Methotrexate

Methotrexate (MTX) is a disease modifying antirheumatic drug, used in 50–70% of patients with rheumatoid arthritis. At low doses, MTX interferes with thymidine and adenosine metabolism leading to an elevation in the extracellular adenosine level. The latter is a potent inhibitor of inflammation via suppressing antigen-dependent immune cell activation and chemotaxis. Besides, MTX is an antimetabolite that inhibits dihydrofolate reductase, a critical enzyme in the de novo synthesis of thymidine, leading to a block in DNA replication and subsequent repression of lymphocytes formation and function [16, 49]. A prospective study by Choi et al. reported that MTX reduces all-cause mortality of rheumatoid arthritis patients, mainly by reducing cardiovascular deaths by 70% [50], though the cardiovascular-protective effect was not confirmed in atherosclerosis patients [51]. Yet, the pharmacological mechanism of MTX remains relevant and is worth investigating in myocarditis. Few experts use MTX in steroids-combined immunosuppression regimens for patients intolerant for AZP or CsA (Fig. 1). So far, there are no published clinical data regarding the efficacy and safety of this regimen.

### Sirolimus

Sirolimus, also called rapamycin, is a relatively new immunosuppressant with anti-proliferative effects due to its ability to inhibit a key protein in cell proliferation, called mammalian target of rapamycin (mTOR), and ultimately inhibits IL-2-mediated T (mainly) and B lymphocyte activation [16, 52]. Moreover, sirolimus was shown to reduce inflammation via the reduction of IL-6 formation [53]. Thanks to its anti-proliferative properties, sirolimus-eluting coronary stents are used in clinical practice to prevent restenosis [54, 55]. mTOR was shown to be involved in cardiac inflammation via NF-kB signaling, whereas its inhibition may modify cardiac inflammation and hypertrophy [56]. Several evidences supporting the potential of mTOR-inhibition as a strategy against cardiomyopathy were reviewed by Kuschwaha et al. [57]. Recently, an in silico drug repositioning study [58] has identified sirolimus as a potential therapeutic option for Infl-CM, based on a systemic comparison of drug-induced gene expression profiles from twelve patients. However, the computational approach was limited by the very small sample size. In cell culture experiments, sirolimus has been shown to promote CVB3-induced cytopathic effects and apoptosis [59]. This highlights that the use of sirolimus in virus-positive myocarditis could be hazardous. Currently, there are no sufficient clinical data advocating the off-label use of sirolimus in myocardial inflammation.

## Strategy II: Systemic Modulation of the Immune Response

An alternative approach to systemic immune suppression is the regulatory adjustment of specific immune and inflammatory responses by the action of an immunomodulator. In this section, the use of different immunomodulatory strategies in Infl-CM is overviewed.

### Intravenous Immunoglobulins

Human intravenous immunoglobulins (IVIGs) are unspecific polyclonal antibodies extracted from pooled human sera. IVIGs have normalizing effects on the host immune system mediated via inhibiting pro-inflammatory cytokines, inducing anti-inflammatory cytokines, expanding regulatory T cells (T_reg_), and blocking pathogenic antibodies via idiotypic-anti-idiotypic interactions and Fc receptor binding. In addition, IVIGs therapy aids to clear pathogens via pathogen opsonization, complement, and effector cell activation [60–63]. IVIGs provide an immunomodulatory therapeutic option for several autoimmune and inflammatory disorders [64–66]. The therapeutic utility of IVIGs in Infl-CM is controversial. On the one hand, the IMAC (Intervention in Myocarditis and Acute Cardiomyopathy) study could not proof the benefit of IVIGs treatment in acute myocarditis patients [67], whereas it seems particularly effective for the treatment of neonates with fulminant enteroviral infection [68]. Furthermore, small-scale studies and patient registries showed positive treatment outcomes including clearance of inflammation even in virus-associated myocarditis. Yet virus eradication was imperfect [69–73]. A double-blind placebo-controlled study on B19V cardiomyopathy patients, intended to disentangle the current knowledge, has been completed [NTC00892112].

### Interferon-ß

IFN-β is an endogenous cytokine carrying immunomodulatory and antiviral functions attributed to augmenting antigen presentation and immune cell activation [74, 75]. The clinical value of IFN-β in Infl-CM has been related to viral etiologies, in particular CVB3 and adenovirus, where immunosuppression is contraindicated [5]. Clinical studies employing 6-month IFN-β regimen have been associated with myocardial CVB3 and adenovirus clearance, reduced myocardial CD3+ T cell infiltration, and clinical improvement [76, 77].

### Autoantibody Therapies

Immune adsorption, classically used to remove autoantibodies from blood, has also been investigated in DCM patients with cardiodepressant autoantibodies [72, 78–80]. Immune adsorption therapy is under current investigation in clinical studies and so far considered for both virus-positive/virus-negative patients refractory to systemic immunosuppression. Alternatively, autoantibodies can be neutralized through the intravenous application of small soluble molecules, including peptides or aptamers [81].

### Cell-Based Therapies

T_reg_ are a subpopulation of CD4+ cells that function to maintain immune tolerance and prevent autoimmunity. T_reg_ cells have been shown to be dysregulated and in imbalance with pro-inflammatory Th_17_ cells, in myocarditis and autoimmune DCM [82–85]. Reestablishing the quality and the quantity of the T_reg_ population via direct application into the circulation has been shown to be a successful therapeutic strategy in a murine model of CVB3-induced myocarditis [82, 85, 86].

Mesenchymal stromal cells (MSCs) are plastic-adherent cells that can be isolated from bone marrow and adipose and other body tissues. MSCs are immunoprivileged, exert immunomodulatory properties [87], and are able to home to sites of inflammation, offering therapeutic potential for myocarditis [88, 89]. In a murine CVB3-induced myocarditis model, MSCs have been described to increase circulating T_reg_ cells [90], modulate monocyte trafficking [9], and inhibit activation of nucleotide oligomerization domain (NOD)-, leucine-rich repeat (LRR)- and pyrin domain containing protein 3 (NLRP3) [91] among other cardioprotective effects. Similar to MSCs, cardiac stromal cells derived from endomyocardial biopsies exert immunomodulatory [92] and cardioprotective [93] effects. Their potential to increase T_reg_ cells, to decline cardiac mononuclear cell activity, and to improve LV function has been shown in an experimental model of CVB3-induced myocarditis [94].

### Cannabidiol

Cannabidiol (CBD) is a non-psychoactive cannabis extract, currently under investigation in numerous indications owing to the anti-inflammatory, antioxidant, and cytoprotective effects, and it exerts independent of classical G protein coupled cannabinoid receptors activation [95, 96]. Addressing cardiomyopathies, CBD has been shown to offer cardioprotective effects in diabetic cardiomyopathy and in doxorubicin-induced cardiomyopathy [96, 97]. In a chronic autoimmune myocarditis murine model, Lee et al. have demonstrated that long-term CBD treatment could attenuate T cell responses, oxidative stress, and fibrosis [98]. The anti-inflammatory/immunomodulatory effects of CBD are likely to be beneficial in other forms of myocarditis.

## Strategy III: Antagonizing Key Inflammatory Components

**Fig. 2:**
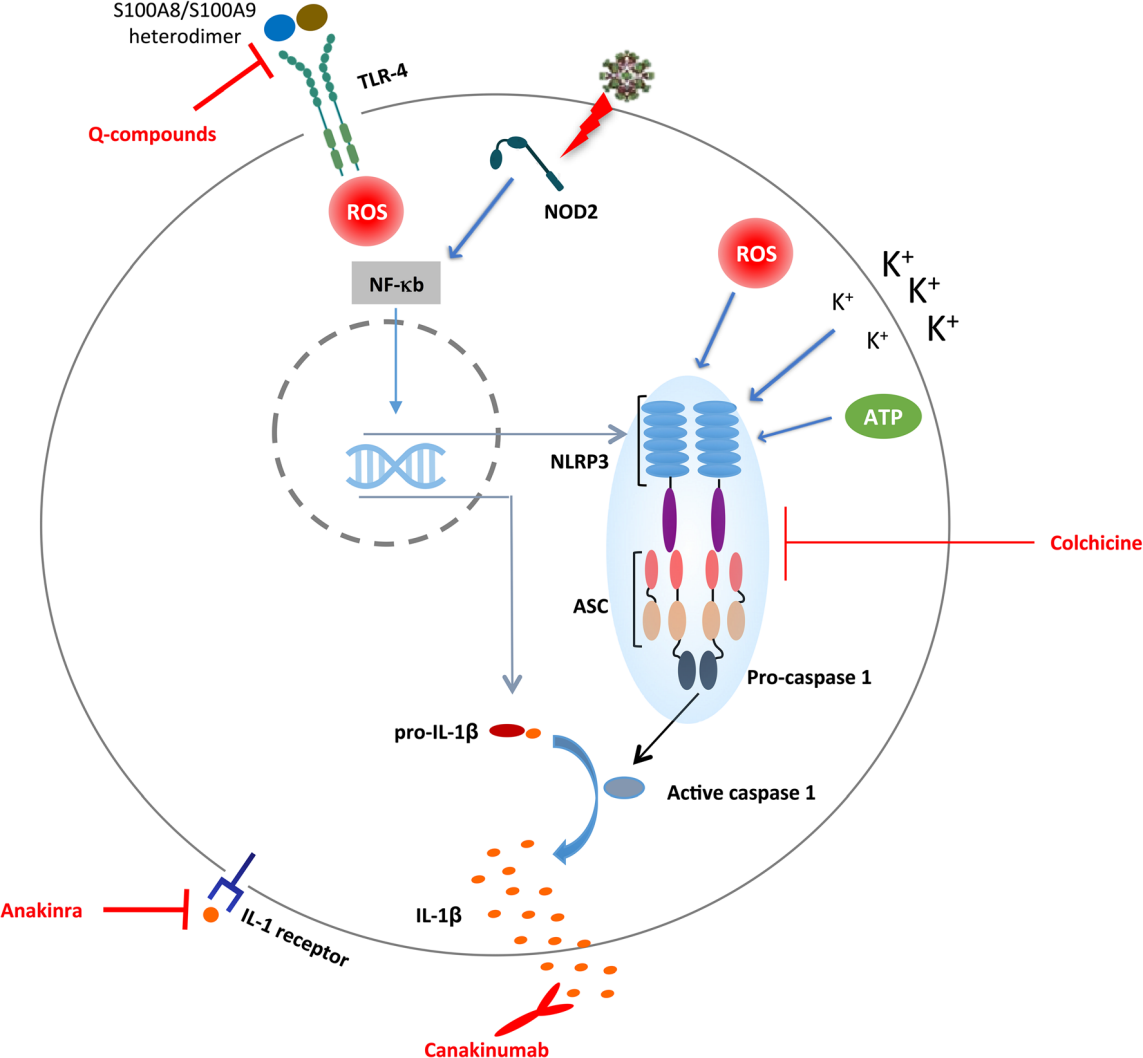
Strategies for antagonizing NLRP3 pathway myocarditis-related key inflammatory components. (1) Upon binding of the S100A8/S1009 heterodimer to the Toll-like receptor 4 (TLR 4) or receptor for advanced glycation end product (RAGE) on an innate immune cell, reactive oxygen species (ROS) are generated in the cytosol, which leads to the activation and translocation of the nuclear factor kappa B (NF-κB) to the nucleus. Alternatively, viral single-stranded RNA, as by coxsackievirus B3 (CVB3), can activate the intracellular receptor nucleotide-binding oligomerization domain-containing protein 2 (NOD-2), which also activates NF-κB. The latter acts as transcription factor that stimulates the mRNA expression of the nucleotide the oligomerization domain-containing, leucine-rich repeat-containing, and pyrin domain-containing protein (NLRP3) inflammasome and of pro-IL-1ß. (2) Upon activation by K+ efflux, ATP, ROS, and other damage-associated molecular patterns, NLRP3 polymerizes with the adaptor protein ASC and caspase 1. Subsequently, caspase 1 cleaves pro-IL-1ß to its active form: IL-1ß. (3) The active cytokine IL-1ß is secreted to the extracellular space, where it binds to its receptor and induces autocrine and paracrine signaling on other immune cells. Q-compounds block step (1) upstream; colchicine blocks step (2); canakinumab and anakinra block step (3) via different mechanisms

Nonsteroidal anti-inflammatory drugs (NSAIDs) are designed to abrogate the synthesis of prostaglandins—a class of eicosanoids—by inhibiting the COX isoforms, responsible for the synthesis of prostaglandins from arachidonic acid. Yet the use of NSAIDs is of limited long-term utility in cardiovascular disorders since they also mediate salt and water retention, and thrombus formation (COX-II inhibitors) worsening heart failure, and therefore not recommended for myocarditis treatment [99, 100]. The present paragraph resumes emerging anti-inflammatory agents for myocarditis or Infl-CM (Fig. 2).

### Antagonizing Neutrophils and NLRP3/IL-1ß Pathway via Colchicine

Colchicine is an anti-inflammatory alkaloid, traditionally used for treatment of and prophylaxis against acute gouty arthritis [101]. The alkaloid interferes with microtubule polymerization, which disrupts the cytoskeleton and arrests mitosis in all rapidly dividing cells. The impact of colchicine on the cytoskeleton critically affects neutrophils functions including chemotaxis, adhesion, and motility. In addition, colchicine reduces superoxide production and inhibits the NLRP3 inflammasome and IL-1β formation (Fig. 2). Together with innate immunity modulation, colchicine exerts anti-inflammatory, anti-fibrotic, and endothelial protective effects [101–103]. The NLRP3 inflammasome, which can be activated via pathogen structures or sterile stimuli, has been identified as inflammatory mediator in myocarditis [91, 104, 105], escorting poor long-term outcomes [106]. In a murine myocarditis model, colchicine abrogated myocardial inflammation, linked with splenic NLRP3 reduction [107]. In clinical studies, colchicine demonstrated favorable treatment outcomes in coronary artery disease [108], pericarditis [109–111], and post-pericardiotomy syndrome [112]. Pericarditis, which is frequently associated with myocarditis, is often treated with colchicine under the recommendation of the most recent ESC guidelines [113]. Since the inflammatory processes and the role of cardiotropic viruses do not differ, in the most cases, between peri- and myocarditis, colchicine is suggested to be effective in myocarditis/Infl-CM, too. In line, a case report has indicated the efficacy of colchicine to treat patients with myocarditis [114].

### Antagonizing IL-1 via Anakinra and Canakinumab

NLRP3 inflammasome activation culminates—through several steps—into the proteolytic cleavage of pro-IL-1 and pro-IL-18 to the active cytokine forms IL-1ß and IL-18 [115–117]. Murine model studies described the pivotal role of IL-1ß in the pathogenesis of autoimmune and viral myocarditis, highlighting the therapeutic potential of IL-1ß-blocking agents [118, 119]. Anakinra is a competitive IL-1 receptor antagonist (Fig. 2), approved for rheumatoid arthritis patients, who are not responding to conventional disease modifying agents [120]. Rheumatic disease is commonly associated with extra-articular cardiovascular manifestation. There was early evidence that anakinra treatment can improve cardiac function in acute rheumatoid arthritis patients [121]. Randomized controlled studies further demonstrated the advantageous outcomes of anakinra treatment in serious cardiac conditions including acute myocardial infarction [122], acute decompensated heart failure [123], heart failure with preserved ejection fraction [124], and pericarditis [125]. In two case reports, Cavali et al. described dramatic improvement of fulminant myocarditis derived-cardiogenic shock shortly after initiation of anakinra [126]. At the moment the superiority of anakinra to standard heart failure treatment in acute myocarditis is being investigated in a triple-blind randomized clinical trial [ARAMIS; NCT03018834]. An alternative mechanism is offered by canakinumab (Fig. 2), a monoclonal antibody that binds and neutralizes IL-1ß [16]. The CANTOS trial, which employed canakinumab as an anti-inflammatory strategy in atherosclerotic patients, reported lower rate of cardiovascular events and circulating levels of IL-1ß, IL-6, together with high-sensitivity C-reactive protein [127]. To date, the efficacy of canakinumab in myocarditis treatment is not reported. In contrast to transplant-related immunosuppressant agents, cytokine inhibitors portray a rapid onset of action and a remarkably higher safety profile. Yet, blocking IL-1ß is still associated with a high risk of infection [127].

### Antagonizing S100A8/S100A9 Alarmins via Q-Compounds

S100A8 and S100A9 alarmins are pro-inflammatory proteins secreted by phagocytes during inflammation [128]. In the presence of calcium, S100A8 and S100A9 form heterodimers that bind to Toll-like receptor (TLR) 4 and receptor for advanced glycation end products (RAGE), expressed on the surface of peripheral blood mononuclear cells. TLR 4 and RAGE stimulate the production of reactive oxygen species that act as secondary messengers to activate the transcription factor NF-κB, which in turn—in the presence of ATP—leads to the expression of NLRP3 and pro-IL1ß. The latter is further cleaved via caspase-1 resulting in the secretion of the active cytokine IL1ß [129–131] (Fig. 2). S100A8 and S100A9 have been shown to correlate with different cardiovascular diseases including myocardial infarction and myocarditis [80, 132, 133]. In CVB3-positive myocarditis patients, the expression of S100A8 and S100A9 in EMB specimens was associated with bad prognosis [134]. Experimental immunomodulatory compounds of the quinoline-3-carboxamides family, referred to as Q-compounds, offer a novel pharmacological approach to treat autoimmune/inflammatory diseases like systemic lupus erythematosus via blocking the binding of S100A9 to TLR 4 [135]. Treating specific Infl-CM patients with Q-compounds based on S100A8/S100A9 serum levels seems to be a promising target-directed strategy, worth evaluating in clinical studies [8].

## Strategy IV: Reducing Myocardial Wall Stress via Mechanical Unloading

Myocardial inflammation may lead to contractile dysfunction and provoke hemodynamic compromise and an increase in wall stress [136]. The latter in turn can activate mechano-transduction pathways, which forces a myocardial inflammatory status [137]. Local and systemic activation of the renin angiotensin aldosterone system (RAAS) in overloaded hearts contributes to myocardial inflammation and remodeling [137, 138]. Interestingly, T lymphocytes and macrophages have been shown to respond to mechanical stress [139, 140]. Mechanically induced T lymphocyte activation further drives extracellular matrix deposition and fibrosis [139]. In macrophages, mechanical stress can advance a pro-fibrogenic phenotype [140].

Axial flow pumps like Impella 2.5, CP, and 5.0 devices allow mechanical unloading of the LV, which does not only reduce myocardial work, energy expenditure, and oxygen demand, but also reduce wall stress [136]. In combination with prednisolone/AZP immunosuppression, prolonged Impella (PROPELLA) support performed via an axillary artery implanted impella 5.0 for up to 39 days has been shown to abrogate myocardial expression of S100A8 and S100A9 alarmins, adhesion molecules, and integrins, in line with reduction in immune cell infiltration, enhancing recovery [141]. The effects were abrogated after explantation of the Impella, despite continuation of immunosuppressive therapy, suggesting an unloading-dependent mechanism. The PROPELLA concept and mode-of-action was further confirmed in a HIV-positive patient in cardiogenic shock due to an EMB-proven viral-negative fulminant myocarditis, by which PROPELLA was performed in the absence of immunosuppressive therapy due to preexisting AIDS [141]. Accumulating evidence advocates mechanical unloading as valuable treatment for compromised myocarditis patients. Large-scale clinical studies are necessary to further confirm the disease-modifying actions of PROPELLA beyond mechanical circulatory support.

## Strategy V: Decreasing Mimic Peptides-Driven Anti-Cardiac Autoimmunity via Antibiotic Therapy

Environmental and genetic factors are considered critical determinants of myocarditis pathogenesis [142]. The exposure of normally hidden myocardial antigens following infections can predispose myocarditis in genetically susceptible individuals [143]. Gut microbiota can prime T_H_ cells against bacterial antigens that mimic cardiac antigens like myosin heavy chain. Following subclinical ischemia or infection that allow antigen exposure, cross-reactive microbiota-driven T_H_ cells can promote myocardial damage. [144–146]. Gil-Cruz et al. [146] showed that broad-spectrum antibiotic treatment could dampen T_H_ cell-mediated cardiac inflammatory responses, trigged by commensal *Bacteroides theca* mimic peptides. This might also be of relevance in the context of virus-induced or virus-associated Infl-CM and needs further investigation.

## Discussion

Systemic immunosuppression is the most potent strategy to defeat myocardial inflammation. Theoretically, any combination of immunosuppressant agents that harbor no hazardous interactions can be considered for Infl-CM treatment. The choice of whether to administer immunosuppressive agents should be seen as personalized practice, based on the whole clinical picture including other comorbidities, type of immune infiltrates, and microbes detected in the myocardium. Every regimen has to be weighed against the risk of infection, including reactivation of latent cardiac microbial infections, outburst of present cardiac virulent infections, and novel infections. Corticosteroids are found almost indispensable in every immunosuppressive combination, attributed to their chief anti-inflammatory activity. The dose of steroids required to produce an anti-inflammatory action is lower, compared with the immunosuppressive dose [16, 147]. Combining prednisolone with potent immunosuppressant agents allows steroid dose reduction while preserving the anti-inflammatory mechanism. This approach shields prone patients like diabetics against steroid adverse effects. Clinical experience with the individual immunosuppressive agents is a major factor in the selection of the regimen by the treating physicians, which favors corticosteroids to a large extend. An immunosuppressive regimen should be initiated at low dose with up titration and may be stepped up to include different classes of immunosuppressant agents (Fig. 1), while through blood levels and common side effects need to be real-time monitored. The treatment protocols need to be shaped and synchronously refined based on the treatment associated benefits and risks aiming to achieve the best therapeutic outcome. More specific strategies meant to block specific myocarditis-related inflammatory mechanisms are currently under investigation yet barely used clinically against myocarditis due to the very limited availability of safety and efficacy data. Colchicine, the relatively cheap old-timer drug, can be an alternative for patients intolerant to steroids. Antagonizing the key inflammatory cytokine IL-1ß is a more specific, relatively safe and rapid onset treatment approach. However, combining systemic immunosuppression agents like corticosteroids or MMF with cytokine blocking agents is limited by a high risk of infection. Colchicine as steroid-free option, not associated with global immune suppression side effects, is safer to combine with IL-1ß antagonists. Moreover, the combination provides a dual approach to block the NLRP3 inflammasome pathway. Autoantibody therapies are considered the last line option for Infl-CM treatment, while mechanical unloading approaches seem favorable in acute cardiogenic shock cases.

## Conclusion

Despite the advancements in basic and clinical research, Infl-CM remains to be an unmet medical need. The different immunosuppressive and anti-inflammatory strategies described in this review are based on single-center experiences and small-scale studies. Treatment of myocarditis is mainly based on the off-label use of major systemic immunosuppressive agents, associated with numerous side effects. Several therapeutic targets revealed by basic research still need to be translated to the bedside. Large-scale randomized placebo-controlled clinical studies are of absolute necessity to designate safe and effective treatments.

## Abbreviations

AZP: Azathioprine
B19V: Parvovirus B19
CBD: Cannabidiol
CCL: CC-chemokine ligand
CsA: Cyclosporine A
COX: Cyclooxygenase
CVB3: Coxsackievirus B3
DCM: Dilated cardiomyopathy
EF: Ejection fraction
EMB: Endomyocardial biopsy
IFN: Interferon
IL: Interleukin
Infl-CM: Inflammatory cardiomyopathy
LV: Left ventricular/left ventricle
mTOR: Mammalian target of rapamycin
MTX: Methotrexate
NF-κB: Nuclear factor-κB
NLRP3: Nucleotide oligomerization domain (NOD)-, leucine-rich repeat (LRR)-, and pyrin domain-containing protein 3
NSAIDs: Nonsteroidal anti-inflammatory drugs
RAAS: Renin angiotensin aldosterone system
ROS: Reactive oxygen species
RTX: Rituximab
TNF: Tumor necrosis factor
T_reg_: Regulatory T cells
